# PB.46: Peutz-Jeghers Syndrome and carcinoma of the breast: call for new breast imaging surveillance guidelines

**DOI:** 10.1186/bcr3546

**Published:** 2013-11-08

**Authors:** FJ Tsang, S Mallappa, W Teh, SK Clark

**Affiliations:** 1 Northwick Park and St Mark's Hospitals, Northwest London Hospitals NHS Trust, London, UK

## Introduction

Peutz-Jeghers Syndrome (PJS) is a rare autosomal dominantly inherited polyposis syndrome caused by STK11 germline mutation. PJS is characterised by gastrointestinal polyps and mucocutaneous pigmentation. These patients have an increased risk of developing cancers, luminal gastrointestinal cancers and breast cancer being the most common, then pancreatic cancer [1,2].

## Methods

A retrospective review of a prospectively maintained database at a tertiary referral centre was carried out. A total of 136 patients from 92 families were included.

## Results

The median age when patients were first seen was 20 years (range, 2 to 65). Twenty-seven carcinomas were detected in 19 patients; 15 were carcinoma of the breast (55%) (two patients had bilateral disease). All of the breast cancers were in women and were predominantly ductal carcinoma *in situ*. Of the 13 patients with breast cancer, a STK11 mutation was detected in eight (62%).

## Conclusion

The cumulative risk of developing breast cancer is 31 to 54% at age 60 years, with a median age at diagnosis of 37 years (range, 19 to 48 years) in PJS patients [3]. Published European guidelines recommend annual magnetic resonance imaging of the breast from age 25 to 30 years, with mammography being substituted after age 50 years [3], which we endorse.
